# Correlation of Tissue Biopsy and Fine Needle Aspiration Cytology with Positron Emission Tomography Results

**DOI:** 10.4061/2011/323051

**Published:** 2011-04-06

**Authors:** Daniel Rosen, Bruce Herrington, Peeyush Bhargava, Rodolfo Laucirica, Gordana Verstovsek

**Affiliations:** ^1^Department of Pathology, Baylor College of Medicine, Houston, TX 77030, USA; ^2^Department of Radiology, Michael E. DeBakey VA Medical Center, Baylor College of Medicine, Houston, TX 77030, USA; ^3^Department of Pathology, Ben Taub General Hospital, Baylor College of Medicine, Houston, TX 77030, USA; ^4^Department of Pathology, Michael E. DeBakey VA Medical Center, Baylor College of Medicine, 2002 Holcombe Boulevard, Houston, TX 77030, USA

## Abstract

F-18-fluorodeoxyglucose (FDG) Positron Emission Tomography (PET) scans are positive in any condition which increases metabolism in a mass or tissue and are therefore not specific for neoplastic conditions. The use of an SUV cutoff value of 2.5 may not always help discriminate between benign and malignant cases. For a practicing cytopathologist doing adequacy checks during an image-guided procedure, it may be of value to be aware that elevated SUV values are not always indicative of a malignant process, and vice versa.

## 1. Introduction

Positron Emission Tomography (PET) is a form of nuclear medicine technology that measures bodily functions, such as blood flow, oxygen use, and glucose metabolism. The procedure utilizes a radioactive “tracer” substance, which is typically injected into the bloodstream. This radioactive material accumulates in organs and decays by the emission of gamma rays. These are captured by a PET scanner, and, with the aid of a computer, an image is generated. Unlike other imaging modalities, PET studies are less directed toward depicting anatomy and structure and more concerned with depicting physiologic processes [1]. This includes the rates of glycolytic metabolism or levels of other various chemical activities that are often high in malignancy. 

A commonly used tracer substance is the glucose analogue, F-18-fluorodeoxyglucose (FDG). Areas of greater intensity, called “hot spots,” indicate where large amounts of the radiotracer have accumulated and where there is a high level of glucose hypermetabolism. Less intense areas, or “cold spots,” indicate a smaller concentration of radiotracer. FDG utilization can be used to semiquantify metabolic activity via the generation of “standardized uptake values,” or SUVs [2]. These “hot” and “cold” spots correlate to higher or lower SUVs, respectively.

PET has found wide-spread application in the field of oncology, where it is used in the differential diagnosis, staging and therapy monitoring of oncologic disease [3]. However, there are limitations to the procedure. Body positioning, as well as movement during the procedure, has been shown to affect the results [4]. Additionally, altered metabolic rates or chemical balances may yield false results. A PET scan may be positive in any condition that results in the elevated metabolism of a mass or tissue. This could include inflammatory states, as well as other benign processes [5]. Therefore, PET is not specific for neoplastic states. If a lesion is identified by a PET scan, it may need to undergo a biopsy to determine benign nature versus malignancy. The reported sensitivity and specificity varies greatly among studies, and, in many instances, there is a lack of histologic confirmation. The correlation of tissue diagnoses with PET scan-identified lesions in our institution is unknown. The aim of our study was to evaluate the overall accuracy of positive PET scans at detecting malignant lesions (i.e., the number of positive PET cases confirmed malignant by tissue diagnosis).

## 2. Study Design

We searched the electronic records of Veteran Affairs Medical Center, Houston, Texas to identify patients that had a fine needle aspiration (FNA) or tissue biopsy performed as a consequence of a PET-positive result, over a twenty-four-month period. Cases where biopsy or FNA procedure preceded the PET scan were not included in the study. “PET impression” was defined as a qualitative evaluation of the visually recognized focal area of hypermetabolism. PET impression was divided into four categories: positive, negative, suspicious, and indeterminate. Positive PET impression was defined as a well-defined focus of abnormal FDG uptake, more active than the surrounding tissue, and with an SUV more than 2.5. Areas with no activity or activity less than that of the adjacent tissue were identified as negative by PET impression [6, 7]. Cases in which there was any FDG uptake qualifying as focal hypermetabolism with SUV less than 2.5 or SUV more than 2.5 but visually not focally hypermetabolic were classified as “suspicious” by PET impression. Indeterminate was any uptake or lesion which could not be classified as above. All PET studies were evaluated for PET impression by one Nuclear Medicine Physician for benignancy versus malignancy blinded to the tissue diagnosis. SUV was measured in the focus of hypermetabolism by drawing the region of interest (ROI) for all cases. SUV of 2.5 or greater is reported to be more indicative of malignant rather than benign lesions [8, 9]. Correlation of tissue diagnoses with the PET impression and the standard uptake value (SUV) using 2.5 as a cutoff was performed and the sensitivity and specificity calculated. Cytology or biopsy specimens obtained from PET-negative results were obtained from patients who had a PET-positive lesion with a concurrent positive biopsy of that site. In addition, some of these patients had biopsies of other locations (that were PET negative) for staging purposes. “PET grouped” refers to the analysis of the sum of both PET impression positive and PET impression suspicious groups. Cases with PET inconclusive interpretation were not included in the specificity or sensitivity analysis.

## 3. Statistical Methods

Basic statistical analysis to calculate percentage, specificity, and sensitivity were done using Microsoft Office Excel 2007. Differences in proportions among SUV value and biopsy results were calculated using Student's *t*-test. Receiver operating characteristic (ROC) curve was used to evaluate SUV and biopsy-FNA results. An ROC curve is a plot of the true positive fraction (sensitivity) versus the false positive fraction (one minus the specificity). ROC curves were constructed for the whole group of cases and controls. The area under the ROC curve (AUC) was also calculated (Statistica Version 8, Statsoft, Tulsa, OK).

## 4. Results

 A total of 1383 biopsies and FNA cytologies were found in our electronic records, of which 95 had tissue and available corresponding preceding PET scan to be included for the final analysis. Most diagnostic procedures resulted in cytology specimens (from fine needle aspirations, *n* = 65); the type of diagnostic procedure is detailed in Table 1, and the organ location, where PET scan and tissue diagnosis were performed, is depicted in Table 2. These 95 procedures were performed in 54 patients, of which 53 were male and 1 female (this reflects usual demographics of a Veterans Affairs hospital, where most patients are male). The average age was 66.5 years at time of diagnosis (42–88 years old). The average time that elapsed between PET scan and diagnostic procedure was 36 days (0–288 days). 

Forty-six (49%) lesions were interpreted as positive on PET scan, of which 37 (80%) were malignant, 8 (18%) benign, and 1 (2%) nondiagnostic on cytology or biopsy. Twenty-four cases (25%) had a negative PET scan, all of which were benign on cytology or biopsy. Twenty-one (22%) lesions were interpreted as suspicious on PET scan, of which 9 (43%) were malignant, 10 (47%) were benign, 1 (5%) rendered nondiagnostic material, and 1 (5%) inconclusive result on cytology or biopsy. A total of 4 cases (4%) were interpreted as inconclusive on PET scan, of which 2 (50%) were diagnosed as positive and 2 (50%) as negative on cytology (Table 3). 

**Figure 1:**
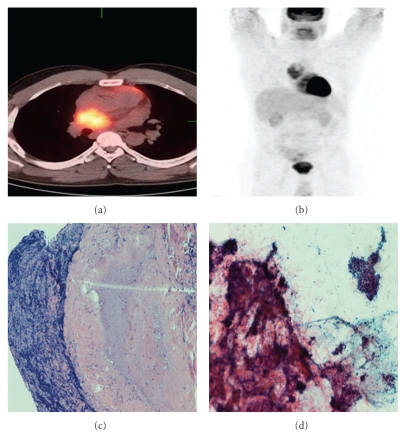
A case showing PET scan diagnosed as “positive for neoplastic process” and a corresponding negative biopsy. (a, b) Intense FDG radiotracer uptake in the mediastinal lymph node. (c) Surgical specimen showing caseating granuloma (H&E 100x). (d) FNA showing absence of malignant cells and clusters of epithelioid cells admixed with lymphocytes and debris (Papanicolaou 100x).

PET-positive/FNA-biopsy negative cases were found in 8 procedures performed on 6 cases. These corresponded to 5 lung lesions and 3 lymph nodes. On pathologic exam, these cases showed either no pathologic change (one case), necrotizing granuloma (one case; Figure 1), or chronic inflammatory changes (three cases). In addition, 10 PET suspicious-biopsy/FNA negative cases were identified showing reactive changes, inflammation, aspiration pneumonia, reactive lymphoid hyperplasia, and a villous adenoma. 

The correlation between SUV and biopsy results is shown in Table 4. A total of 19 cases (32%) with SUV > 2.5 had a negative FNA or biopsy result. These cases were diagnosed as negative for malignancy, reactive lymph nodes, villous adenoma, and necrotizing granuloma. Seven procedures (22%) with an SUV < 2.5 with a positive FNA or biopsy corresponded to 6 patients (Table 5). The first case was a metastatic adenocarcinoma consistent with a pancreatic primary. By immunohistochemistry the tumor cells were cytokeratin 7 positive, cytokeratin 20 negative, and TTF-1 negative. The patient was found to have a 7.3 cm pancreatic head mass by imaging studies. The remaining cases included a poorly differentiated squamous cell carcinoma, basaloid carcinoma of the lung, B-cell lymphoma follicular type, small lymphocytic lymphoma, and poorly differentiated squamous cell carcinoma (Table 5).

**Figure 2:**
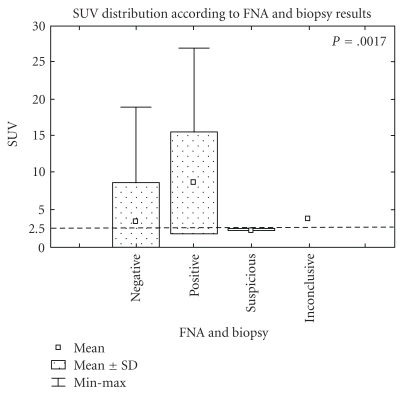
Distribution of SUV according to biopsy result.

**Figure 3:**
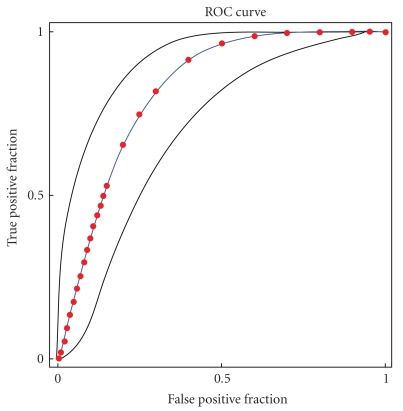
ROC curve.

A box plot showing the SUV distribution according to the biopsy/FNA result is shown in Figure 2. The average SUV for the negative biopsy group was 3.3 (0–18.9, SD: 5.2) and for the positive biopsy group 8.6 (0–26.9, SD: 6.7). There was a significant overlap among negative and positive cases as shown in Figure 2. However, the difference between negative and positive biopsy groups was statistically significant (*P* < .0017). In concordance, the ROC curve shows that an SUV cutoff of 2.5 has a significant discriminatory value (Figure 3). The calculated area under the curve was 82.3%.

The overall sensitivity and specificity for PET impression was 100% and 75%, respectively (Table 6). When biopsy and FNA results are correlated with SUV, the overall sensitivity and specificity were 84% and 52%, respectively (Table 6). Overall, the sensitivity and specificity were higher for PET impression compared to the SUV. When suspicious and inconclusive cases were grouped together with the positive results, the sensitivity dropped, while the specificity remained basically unchanged (Table 6).

## 5. Discussion

In this study we analyzed the correlation between pathology diagnosis (obtained either by FNA or biopsy) and the corresponding PET scan result. We found that the sensitivity and specificity were higher for PET impression (qualitative interpretation of a PET scan which takes into account visual interpretation of FDG uptake coupled with SUV value) compared to the SUV alone (quantitative measure of FDG tracer uptake with a cutoff value of 2.5) [8, 9].

Currently, the application of Positron Emission Tomography (PET) in the diagnosis, staging, and monitoring of therapeutic response has gained wide acceptance in the field of oncology. Metabolism of the most commonly used tracer, F-18-fluorodeoxyglucose (FDG), can be used to semiquantify metabolic activity in tissues of interest via generation of “standardized uptake values,” or SUVs. Standardized uptake values of greater than 2.5 are reported to be more indicative of malignant conditions [8, 9]. However, studies comparing PET impression and SUVs with FNA or biopsy outcomes are sparse. In 2007, Pansare et al. performed a retrospective analysis of PET scan SUV with final FNA results [10]. Using an SUV cutoff of 2.5, their findings showed that, for lesions with an SUV > 2.5, 87% proved to be malignant and 13% benign on tissue diagnosis. Of the lesions with an SUV < 2.5, 54.5% showed benign cytology and 45.5% malignant cytology. The reported sensitivity, specificity, positive predictive values (PPV), and negative predictive value was 84%, 60%, 87%, and 56%, respectively. In comparison to Pansare's findings, the sensitivity and specificity from our study are higher. The different characteristics of the patient population, organ sites, and method of collecting the specimens as well as variation in PET analysis may account for these differences. In particular, the number of true negative and false negative cases may be difficult to obtain. Normally, tissue sites that are negative on PET scan are not biopsied or are biopsied rarely. Therefore, the total number of true negative and false negative cases is difficult to assess and can be variable among studies. In our study, these cases (PET positive/biopsy-FNA negative) were obtained from patients who had a PET-positive lesion with a concurrent positive biopsy of that site, and in addition these same patients had biopsies of other locations (that were PET negative), for staging purposes. The number of these cases was small and may not accurately reflect the true correlation of PET diagnosis and pathologic diagnosis. Other considerations that may contribute to differences among studies are organ site, type of tumor, size of the lesion, and metabolic state of the tumor cells. For example, the reported sensitivity and specificity PET CT of lung lesions is 96% (range: 83–100%) and 79% (range: 52–100%) [11, 12], for colorectal cancer 97% (95–99%) and 75.6% (64–88%) [13, 14], for Hodgkin lymphomas 84% (71–92%) and 90% (84–94%) [15], non-Hodgkin lymphomas 72% (61–82%) and 100% (97–100%) [15], esophageal tumors 51% (27–93%) and 84% (41.7–95.2%) [16, 17], and head and neck tumors 98% (88–100%) and 92% (75–100%), respectively [18–20]. One of the limitations of our study was the enrollment of patients with known history of cancer. Further studies, including more homogeneous and larger cohort of patients stratified by anatomic site and histologic diagnosis are needed to further characterize and define SUV cutoff values for particular organ system in our patient population.

In our study, we also assessed the correlation between PET impression with the final tissue diagnosis. For lesions diagnosed by PET impression as positive, 80% proved malignant and 18% benign on cytology or biopsy. One case (2%) with a PET-positive impression was signed out as nondiagnostic on cytology. All 24 cases that were diagnosed as negative by PET impression were diagnosed as benign on cytology or biopsy. The overall sensitivity and specificity was 100% and 75%, respectively. In comparison to the results found by Pansare, the sensitivity when utilizing PET impression was roughly the same; however, the specificity appears notably higher (75% versus 60%) [10]. 

The SUV threshold of 2.5 has been used in most studies to discriminate benign from malignant lesions [21, 22]. However, receiver operation characteristic analysis has shown that a highest diagnostic accuracy can be achieved using SUV thresholds of 4.4 or higher [23–25]. On the other hand, such high threshold would significantly increase the false negative rates and may have suboptimal clinical impact [26]. According to one study, one can omit surgical staging in patients with a PET-negative mediastinum [27]. Furthermore, in our study, an SUV of 2.5 does not seem to segregate positive and negative cases adequately. Even though the ROC curve analysis showed a significant discriminatory value and cases with a negative biopsy result tend to have a significantly lower SUV (mean 3.3) compared to positive biopsy result (mean 8.2), there was a significant overlap in the overall distribution of the SUV among these two groups as shown on the box plot analysis. Overall, PET impression was more accurate in determining whether a lesion was benign or malignant than the SUV value alone. Some studies have reported the use of different SUV cutoff values to better discriminate benign versus malignant lesion [28]. The use of a single universal SUV cutoff may not always be appropriate. 

Regarding false positive results, eight PET-positive/biopsy-FNA negative cases corresponded to 6 patients with 5 lung lesions and 3 lymph nodes. Two cases showed inflammatory changes, and the remaining 6 cases were diagnosed as negative for malignancy. Benign conditions that cause increased glucose uptake can result in elevated SUV [5]. Nonspecific inflammatory lesions in lymph nodes, as well as various infectious etiologies, have been shown to cause SUV elevation which can sometimes be misleading [5], implying a malignant process. Among others, some examples include sarcoidosis, lymph node with follicular hyperplasia, tuberculosis, histoplasmosis, and aspergillosis [5]. The proposed mechanism responsible for this phenomenon is increased glucose uptake by inflammatory cells (e.g., neutrophils and macrophages) within the lesional tissue [5]. This seems a plausible explanation for our false positive PET results.

In our study we did not encounter any false negative PET studies (PET-negative/biopsy-FNA positive cases). Studies have reported the sensitivity of the PET scan ranging from 50% to over 90% while in our study was 100% [29]. In the literature false negative PET scans have been reported in well-differentiated adenocarcinomas, purportedly due to low glucose metabolism and/or low tumor cell density [5]. Examples of low-grade lesions possibly yielding false negative results via PET could include bronchioalveolar lung carcinoma or small lymphocytic lymphoma. It has also been speculated that this false negative result may be due to the presence of necrosis in high-grade lesions [10]. In addition, organ location, histologic tumor type, metabolic status of the patient, and size of the lesions, among others, may also account for the wide range of reported sensitivity [29]. 

In conclusion, our study indicates that an SUV cutoff value of 2.5 does not always adequately discriminate between malignant and benign processes, as confirmed by follow-up tissue diagnosis. While a negative PET study most likely excludes a malignant process, a positive PET scan may be due to either a malignant process or reactive/inflammatory condition, and therefore it may be useful to undertake further diagnostic attempts (such as FNA) to better define the lesion. For a practicing cytopathologist doing adequacy checks during an image-guided procedure, it may be of value to be aware that elevated SUV values are not always indicative of a malignant process, and vice versa. This, among other factors (such as, but not limited to, cellularity and presence of lesional tissue), may help in determining the number of required passes to get adequate diagnostic material. The observed difference in our findings and other studies highlights the need for additional investigation in this area, especially investigating specific organ systems, specific site, and specific diagnostic categories on a larger number of patients and correlating with PET scan readings.

## Figures and Tables

**Table 1:** Type of diagnostic procedure.

Procedure	Total
Biopsy	30
TBNA	30
CT FNA	22
Superficial FNA	6
US FNA	6
BRUSH/WASH	1

Total	95

FNA: fine needle aspiration, TBNA: transbronchial needle aspiration, CT FNA: computerized tomography guided FNA, US FNA: ultrasound guided FNA, BRUSH/WASH: bronchial brush/wash done during bronchoscopy.

**Table 2:** Organ sites where PET scan and tissue diagnosis were performed.

Site	Total
Lung	36
Lymph node	
Lung	23
Neck	5
Paratracheal	2
Axilla	2
Mediastinum	2
Supraclavicular	1
Groin	1
Bone	
Rib	3
Vertebral	1
Parotid gland	2
Skin	2
Chest wall	1
Colon	1
Epiglottis	1
Esophagus	1
Soft tissue	
Gluteal	1
Supraclavicular	1
Kidney	1
Liver	1
Mediastinum	1
Neck mass	1
Orbit	1
Oropharynx	1
Rectum	1
Minor salivary gland	1
Thyroid	1

Total	95

**Table 3:** Distribution of cases according to PET impression and Bx/FNA result.

	Biopsy-FNA				
PET impression	Positive	Negative	Nondiagnostic	Inconclusive	Total
Positive	37 (80%)	8 (18%)	1 (2%)	0	46 (49%)
Negative	0 (0%)	24 (100%)	0	0	24 (25%)
Suspicious	9 (43%)	10 (47%)	1 (5%)	1 (5%)	21 (22%)
Inconclusive	2 (50%)	2 (50%)	0	0	4 (4%)

Total	51	41	2	1	95

**Table 4:** Distribution of the cases according to PET SUV and Biopsy-FNA result.

	Biopsy				
SUV	Positive	Negative	Suspicious	Inconclusive	Total
>2.5	39 (66%)	19 (32%)	0	1 (2%)	58
<2.5	7 (22%)	21 (68%)	3 (10%)	0	31
Not available	5 (83%)	1 (17%)	0	0	6

Total	51	41	2	1	95

**Table 5:** Clinical characteristics of the 8 biopsy-FNA positive procedures with SUV < 2.5.

Patient	Age	Organ	Procedure	PET impression	SUV	Diagnosis
1	68	Supraclavicular lymph node	US FNA	Inconclusive	1.5	Metastatic adenocarcinoma
2	77	Mediastinal lymph node	TBNA	Suspicious	2.1	Poorly differentiated squamous cell carcinoma
3	77	Lung	TBNA	Suspicious	2.4	Basaloid carcinoma
4	82	Axillary lymph node	CT FNA	Suspicious	2.3	B-cell lymphoma, follicular type
5	42	Neck lymph node	CT FNA	Suspicious	2.1	Small lymphocytic lymphoma
6	55	Paratracheal lymph node	Biopsy	Negative	0	Poorly differentiated squamous cell carcinoma
6		Paratracheal lymph node	Biopsy	Negative	0	Poorly differentiated squamous cell carcinoma
6		Lung	Biopsy	Negative	0	Poorly differentiated squamous cell carcinoma

US FNA: ultrasound guided FNA, TBNA: transbronchial needle aspirate, CT FNA: CT-guided FNA.

**Table 6:** Comparison of the sensitivity and specificity for PET and SUV.

	Pet impression	SUV	PET grouped*
Sensitivity	100%	84%	100%
Specificity	75%	52%	57%

*“PET grouped” refers to the analysis of the sum of both PET impression positive and PET impression suspicious groups.
